# Prevalence of Atrial Fibrillation in Obstructive Sleep Apnea Patients in a Tertiary Care Center

**DOI:** 10.31729/jnma.4812

**Published:** 2020-02-29

**Authors:** Subodh Sagar Dhakal, Asmita Neupane, Mahesh Bhattarai, Dambar Bahadur Karki

**Affiliations:** 1 Department of Internal Medicine, Kathmandu Medical College and Teaching Hospital, Sinamangal, Kathamandu, Nepal; 2 Kathmandu Medical College and Teaching Hospital, Sinamangal, Kathamandu, Nepal

**Keywords:** *atrial fibrillation*, *obstructive sleep apnea*, *prevalence.*

## Abstract

**Introduction:**

Atrial fibrillation is the most common sustained arrhythmias.Recently there has been evidence of higher prevalence of atrial fibrillation in obstructive sleep apnea patients compared to the general population. The aim of this study was to find the prevalence of atrial fibrillation in patients of obstructive sleep apnea in a tertiary care center.

**Methods:**

This descriptive cross-sectional study was done in Om Hospital and Research Centre from January 2016 to 2018 March after ethical clearance.All the patients who were diagnosed to have OSA underwent detailed history taking, cardiovascular examination and underwent electrocardiogram evaluation.All the patients who were referred for sleep study underwent technician attendant in hospital Level A polysomnography were included. Convenience sampling was done. Data was collected and entry was done in microsoft excel, point estimate at 95% Confidence Interval was calculated along with frequency and proportion for binary data.

**Results:**

The prevalence of atrial fibrillation in patients with obstructive sleep apnea is 7 (10.44%) at 95% Confidence Interval (6.70-14.17%). Apnoea-Hypopnoea Index of more than 30was present in 3 (42.8%) patients of atrial fibrillation. Atrial fibrillation was seen highest, 3 (42.8%) in patients with BMI more than 30 and lowest, 1 (14.28%) patients with BMI less than 23.5. Prevalence of atrial fibrillation was seen 5 (71.4%) in male patients and 2 (28.57%) in female patients. Sixty seven (75.28%) patients had obstructive sleep apnea in which male patients was predominant 48 (71.64%).

**Conclusions:**

Prevalence of atrial fibrillation in patients of obstructive sleep apnea was found to higher than the similar studies done. It is important to obtain detail cardiac history in any patients with obstructive sleep apnea and look for arrhythmias speciallyatrial fibrillation.

## INTRODUCTION

Obstructive sleep apnea (OSA) is a common breathing disorder characterized by recurrent episodes of airway collapse resulting in occlusion of airflow during sleep.^1^ OSA affects upto 25% of middle aged adults.^2^ OSA is a important health problem with a high prevalence and tremendous health care cost.^3^ Atrialfibrillation (AF) on the other hand is the most common arrhythmia responsible for one third of all the arrhythmia related hospitalization.^4^

Sleep apnea has been implicated in the pathogenesis of arrhythmias, hypertension, heart failure and stroke.^5-9^ This can be attributed to a higher prevalence of traditional risk factors for AF like obesity and hypertension in OSA patients, never the less the relation between AF and sleep apnea is found to be stronger than that of sleep apnea and traditional risk factor of AF.^5^

As studiesfrom Nepal are not much present related to OSA and AF, the aim of the study was to find prevalence of atrial fibrillation in patients of obstructive sleep apnea in Om hospital and Research Center.

## METHODS

This descriptive cross-sectional study was done in Om Hospital and Research Centre from January 2016 to 2018 March after ethical clearance from research department from OM hospital with reference number 112,76,77.

All the patients who were referred for sleep study underwent technician attendant in hospital polysomnography in Om Hospital Research Centre from January 2016 to 2018 March. Study population were the patients with obstructive sleep apnea who have been diagnosed with the help of polysomnography. OSA was diagnosed on the basis of Apnoea-Hypopnoea Index (AHI)more than 5.Mild OSA is defined asAHI5-15,moderate 15-30 and severe more than 30.^10^ Inclusion criteria for the study was the patients who were diagnosed to have obstructive sleep apnea at the study site. Exclusion criteria for the participation in study were patients who were already diagnosed to have atrial fibrillation before the study and receiving treatment. All the patients who were diagnosed to have OSA underwent detailed history taking, cardiovascular examination and underwent electrocardiogram evaluation. Absence of P wave along with irregularly irregular rhythm were diagnosed to have AF.^11^

Convenience sampling was done and minimum sample size was calculated as,

n = z^2^ × (p × q)/e^2^

   = 1.96 × (0.03 × 0.97)/0.06^2^

   = 31.04

   = 32

Where,

n = minimum sample size
p = prevalence of atrial fibrillation in OSA patients, 3%.^12^
q = 1-p
e = margin of error, 6%

Since the convenience sampling is done, we have taken the total sample for the study as 67. Therefore, the total sample taken for the study was 67.

Bias present in the study such as selection bias and interviewer’s bias were minimized as possible.

All the data were collected and entry was done in Microsoft excel. Point estimate at 95% CI was done along with frequency and proportion for binary data.

## RESULTS

Among 67 patients who were diagnosed to have OSA after polysomnography, the prevalence of atrial fibrillation was 7 (10.44%) at 95% Confidence Interval (6.70-14.17%). Atrial fibrillation were seen only in OSA patients in our study.Stop Bang questionnaire were used to evaluate the probability of having obstructive sleep apnea along with detailed medical history and clinical examination. Among the seven patients, AHI more than 30 was present in 3 (42.8%) patients (Table 1).

**Table 1:** AHI in patients with atrial fibrillation.

AHI	n (%)
AHI < 5	None
AHI 5-15	2 (28.57)
AHI 5-30	2 (28.57)
AHI > 30	3 (42.8)

As Body Mass Index (BMI) of patients increase, the prevalence of atrial fibrillation was also seen to increase in our study. Atrial fibrillation was seen highest, 3 (42.8%) in patients with BMI more than 30 and lowest, 1 (14.28%) patients with BMI less than 23.5 (Table 2).

**Table 2:** Atrial fibrillation in different BMI group.

BMI	n (%)
< 23.5	1(14.28)
> 23.5-30	3 (42.8)
> 30	3 (42.8)

Among the total atrial fibrillation, prevalence of atrial fibrillation was seen 5 (71.4%) in male patients and 2 (28.57%) in female patients; atrial fibrillation was seen to be more common in male population than in female population (Table 3).

**Table 3:** Gender wise distribution of atrial fibrillation.

Atrial fibrillation	n (%)
Male	5 (71.4)
Female	2 (28.57)

Among the 67 (75.28%) patients with OSA, OSA was most commonly seen in patients of 30-60 age group, 29 (43.28%) followed by 27 (40.29%) patients in age group more than 60 years. OSA was seen predominantly high in male patients with 48 (71.64%) compared to female patients 19 (28.35%). Among the patients with BMI of 23.5-30, OSA was seen highest 44 (65.67%) when compared to patients with other BMI groups (Table 4).

**Table 4:** Baseline characteristic of OSA patients.

Age	n (%)
< 40	11 (16.41)
30-60	29 (43.28)
> 60	27 (40.29)
**Sex**	
Male	48 (71.64)
Female	19 (28.35)
**BMI**	
< 23.5	10 (14.92)
> 23.5-30	44 (65.67)
> 30	13 (19.40)

Among the OSA patients, severe OSA, when AHI more than 30, was seen in 21 (23.59%) patients whereas mild OSA, AHI between 5-15, was seen in 24 (26.96%) patients (Table 5).

**Table 5:** AHI Index among the OSA patients.

AHI	n (%)
AHI 5-15	24(26.96)
AHI 5-30	22 (24.71)
AHI > 30	21(23.59)

## DISCUSSION

In our study, the prevalence of atrial fibrillation was 10.44% in OSA patients which is higher andwas more prevalent in severe OSA when compared to the study done by Guilleminault,et al. which mentioned prevalence to be 3% in OSA patients. In our study, fewer females 28.35% were found to be suffering from OSA and fewer female patients were referred for the sleep study. This might be due to the fact that sleep apnea is noted high in men than women globally. Most of our study population were between the age of 40-60 years and which is consistent with findings of study done by Young T, et al. and Punjabi NM.^2,13^

A large number of patients were obese, this might be due to fact that OSA often go undiagnosed though physician understand the algorithms for the diagnosis of sleep apnea, the majority are unable to identify the patients for whom diagnostics are needed.^14^ Most of the common symptoms presented by the patients were snoring and witnessed apnea which was major symptoms to be reported by the patient’s partner.^15^

In our study obstructive sleep apnea was more frequent than central sleep apnea and all cases of atrial fibrillation were only observed in the patients with OSA. In multiple studies, AF is substantially seen to be more prevalent in patients with OSA than those without OSA which is similar to findings of our study.^5,6^ Potential link between OSA and AF at first from an observational study that reported AF among 3% of subjects with OSA. In these study patients having paroxysmal AF were completely cured after receiving treatment with OSA.^12^ The similar findings have been confirmed further by other studies done by Hoffstein V, et al.^16^ Sleep Heart Health Study reported four times higher prevalence of AF in patients with sleep apnea than patients without sleep apnea.^17^ The association remains strong and significant even after adjustment of covariates including hypertension, BMI.^18^ Since our study was done in a single tertiary care center, the results so obtained cannot be generalized to the general population. Since our study being cross-sectional, it only enables us for point estimate of disease however unable to determine any associations or incidence of disease.

## CONCLUSIONS

Prevalence of atrial fibrillation in patients of obstructive sleep apnea was found to higher than the similar studies done. It is important to obtain detail cardiac history in any patients with obstructive sleep apnea and look for arrhythmias especially atrial fibrillation. As most studies show an increased prevalence of cardiac arrhythmias, in particular, AF in OSA patients and because of its modifiable and high prevalence makes comprehensive cardiac evaluation mandatory in all the patients presenting with OSA. Patients with OSA should be screened for atrial fibrillation and therapeutic interventions for should be given to patients.

## Conflicts of Interest:

None.
